# Neuromodulation of the Autonomic Nervous System in Chronic Low Back Pain: A Randomized, Controlled, Crossover Clinical Trial

**DOI:** 10.3390/biomedicines11061551

**Published:** 2023-05-26

**Authors:** Rob Sillevis, Juan Nicolás Cuenca-Zaldívar, Samuel Fernández-Carnero, Beatriz García-Haba, Eleuterio A. Sánchez Romero, Francisco Selva-Sarzo

**Affiliations:** 1 Department of Rehabilitation Sciences, Florida Gulf Coast University, Fort Myers, FL 33965, USA; rsillevis@fgcu.edu; 2 Universidad de Alcalá, Facultad de Enfermería y Fisioterapia, Departamento de Fisioterapia, Grupo de Investigación en Fisioterapia y Dolor, 28801 Alcalá de Henares, Spain; 3 Research Group in Nursing and Health Care, Puerta de Hierro Health Research Institute-Segovia de Arana (IDIPHISA), 28222 Majadahonda, Spain; 4 Physical Therapy Unit, Primary Health Care Center “El Abajón”, 28231 Las Rozas de Madrid, Spain; 5 Interdisciplinary Group on Musculoskeletal Disorders, Faculty of Sport Sciences, Universidad Europea de Madrid, 28670 Villaviciosa de Odón, Spain; 6 Francisco Selva Physiotherapy Clinic, 46008 Valencia, Spain; beagih@hotmail.com; 7 Department of Physiotherapy, Faculty of Sport Sciences, Universidad Europea de Madrid, 28670 Villaviciosa de Odón, Spain; 8 Physiotherapy and Orofacial Pain Working Group, Sociedad Española de Disfunción Craneomandibular y Dolor Orofacial (SEDCYDO), 28009 Madrid, Spain; 9 Musculoskeletal Pain and Motor Control Research Group, Faculty of Sport Sciences, Universidad Europea de Madrid, 28670 Villaviciosa de Odón, Spain; 10 Department of Physiotherapy, Faculty of Health Sciences, Universidad Europea de Canarias, 38300 Santa Cruz de Tenerife, Spain; 11 Musculoskeletal Pain and Motor Control Research Group, Faculty of Health Sciences, Universidad Europea de Canarias, 38300 Santa Cruz de Tenerife, Spain; 12 Physiotherapy Faculty, Universitat de València, 46010 Valencia, Spain; paco.selva@uv.es

**Keywords:** neuromodulation therapy, transcutaneous, autonomic nervous system, low back pain

## Abstract

Chronic pain is a societal concern influencing the autonomic nervous system. This system can be captured with automated pupillometry. The direct connection between the epidermal cells and the brain is presented as part of the central nervous system, reflecting the modulation of the autonomic system. This study’s aim was to investigate if tape containing magnetic particles (TCMP) has an immediate effect on the autonomic nervous system (ANS) and influences chronic low back pain. Twenty-three subjects completed this study. Subjects were randomized to either receive the control tape (CT) or TCMP first. Each subject underwent a pain provocative pressure test on the spinous process, followed by the skin pinch test and automated pupillometry. Next, the TCMP/control tape was applied. After tape removal, a second provocative spinous process pressure test and skin pinch test were performed. Subjects returned for a second testing day to receive the other tape application. The results demonstrate that TCMP had an immediate significant effect on the autonomic nervous system and resulted in decreased chronic lower back pain. We postulate that this modulation by TCMP s has an immediate effect on the autonomic system and reducing perceived pain, opening a large field of future research.

## 1. Introduction

Lower back pain is a common issue affecting a significant proportion of the population, with lifetime prevalence rates reported greater than 20% [1,2]. Chronic pain, which can involve both peripheral and central mechanisms, affects around 20–25% of people [3]. An injury can cause tissue destruction or damage, activating peripheral nociceptors [4,5,6]. Chronic nociceptive information leads to sensitization, which affects the spinal cord, resulting in increased nociceptive information reaching the brain and resulting in increased central sensitization [7,8,9]. Chronic pain leads to prolonged increased sympathetic activity in the autonomic nervous system (ANS), which can cause disorders such as vagal nerve dysfunction, hyperhidrosis, urinary disorders, orthostatic hypotension, gastrointestinal symptoms, sexual dysfunction and pain [10,11,12,13].

Previous studies have demonstrated that the pupil diameter is a direct reflection of ANS activity and can be measured to obtain a direct impression of ANS functioning [14,15]. Several structures, such as the pupil, are exclusively innervated by the ANS. Based on this, the pupil diameter would be a direct reflection of ANS activity and can be measured [16,17,18,19]. Therefore, the pupil diameter at any given time is a reflection of the real-time balance between the sympathetic and parasympathetic system [5,20,21]. Previous studies have demonstrated the validity and reliability using a method of fully automated pupillometry to capture the pupil diameter effectively in real-time, and thus can be used to obtain a direct impression of the functioning of the ANS [22,23,24].

The skin, hypodermis, and superficial fascia are innervated by the cutaneous branches of individual spinal nerves [25]. The epidermis, which embryologically arises from the ectoderm similar to the brain, does not display a dermatome map as seen in conditions such as radiculopathy. However, treating epidermal dysfunctions such as painful scars could modulate pain in areas away from the intervention site. The interconnectivity between the skin and other systems such as the brain and immune system has been identified [26].

Keratinocytes, which are the most common cells of the epidermis, contain multiple sensory systems that detect environmental changes and neurotransmitters and receptors that play crucial roles in the brain [27]. Recent studies have shown that the excitation of keratinocytes can induce sensory perception in the brain [27]. Cutaneous sensory stimuli are transduced in the periphery by specialized organs or cutaneous nerve endings [28]. The tactile sensory ability in the epidermis is provided by keratinocytes, Merkel cells and free nerve endings [29].

The identification of keratinocytes as the primary transducers of harmful stimuli is a paradigm shift in the field of cutaneous sensory transduction [30]. It has been shown that a low-frequency magnetic field induces the differentiation of HaCaT cells, and the application of a tape containing magnetic particles (TCMP) on the epidermis had an immediate impact on lower back pain and lower limb vascularization [27,28,29,30,31,32,33]. Superficial neuromodulation in the epidermis could result in a measurable change in ANS activity and a decrease in perceived musculoskeletal pain. This would imply that TCMP produces a systemic effect by directly influencing the central nervous system-epidermis connection.

Magnetic particles have potential for improving conventional therapeutic procedures and clinical diagnostics, providing novel biomedicine approaches [34]. Cardoso et al. [34] report that magnetic particles can be specifically designed for the prevention, diagnosis and treatment of diseases. Combining magnetic particles with polymeric biomaterials has shown great potential for tissue repair, such as bone, muscle, nerve and cardiac tissue regeneration [35]. A created magnetic field acts as a vehicle to induce the flow of ions without the direct stimulation of the nerve tissue itself [36]. However, once the ionic flow is created in the epidermal cells, the effect of either electrical or magnetic stimulation at the neural level will be the same. It will produce an axon depolarization and the initiation of an action potential [36].

The particles in the TCMP do not exhibit any magnetization unless they are in direct contact with an external magnetic field. The epidermis has been shown to have a magnetic field, and thus one could hypothesize that the TCMP affects the magnetic field of the epidermis. This could imply that the application of magnetic particles to the epidermis would provide a non-invasive, safe and easy method to treat the area of pain directly. Additionally, it could reduce health care expenditures for managing chronic musculoskeletal disorders [37,38].

To evaluate the immediate effect of magnetic particles using TCMP in subjects with lower back pain, this study’s aim was twofold. The primary aim was to investigate if paravertebral applied tape with magnetic particles in the low back region had an immediate systemic effect on the ANS measured with a method of fully automated pupillometry. The secondary aim was to investigate if the application of this TCMP resulted in an immediate change in local generated pain with posterior to anterior spinous process pressure and the segmental skin pinch test.

## 2. Materials and Methods

### 2.1. Subjects

**Figure 1:**
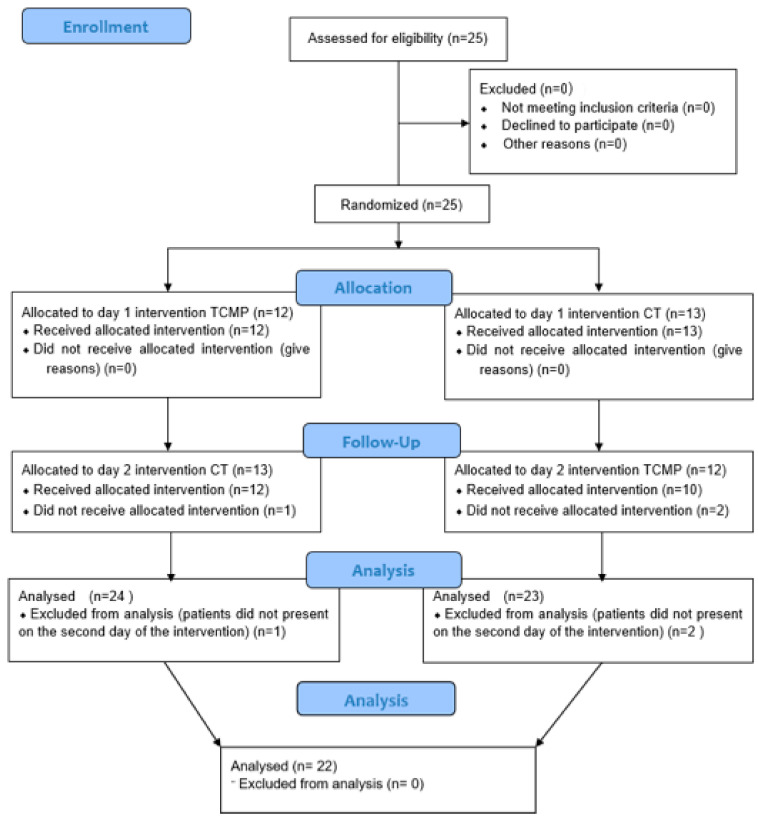
CONSORT flow diagram for dropouts and sample management. All subjects were exposed to all tapes, and three did not finish the study. TCMP, tape containing magnetic particles; CT, control tape.

A total of 25 subjects were recruited using a method of convenience sampling. Three subjects did not show up for the second round of measures, leaving 22 subjects who completed the measuring protocol (Figure 1).

All available patients were screened for inclusion criteria. To participate, all patients had to be: between the ages of 18 and 65, with a medical diagnosis of non-specific low back pain for more than three months of duration of idiopathic origin, able to speak and read the Spanish language fluently, have chronic lower back pain (longer than 3 months, with at least a score of 5 on the Roland-Morris questionnaire), and experience pain at the time of testing (with an numeral pain rating score of 5) [39]. All subjects were screened for any red flags and potential reasons why they could not undergo the testing protocol by the primary investigator. The exclusion criteria encompassed previous spinal surgery and evidence of central nervous system involvement, including hyperreflexia, nystagmus, loss of visual acuity, an impaired sensation of the face, altered taste and the presence of pathological reflexes. Additionally, any diagnosed autonomic disease, central nervous system damage and retinal disease were also exclusion criteria, as they would impact the ANS normal functioning. This study received approval from the University of Valencia with the number 1240878, Spain, and was registered at clinicaltrials.gov (NCT 05504369). All subjects provided written consent before participating in the study.

### 2.2. Randomization of the Sample

The randomization program, Research Randomizer (Version 4.0), https://www.randomizer.org was used.

### 2.3. Automated Measures

**Figure 2:**
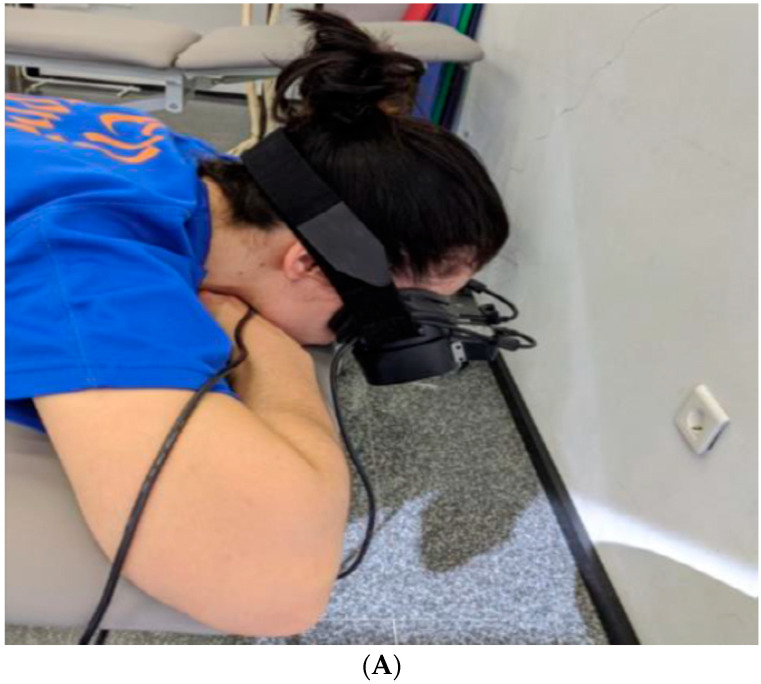

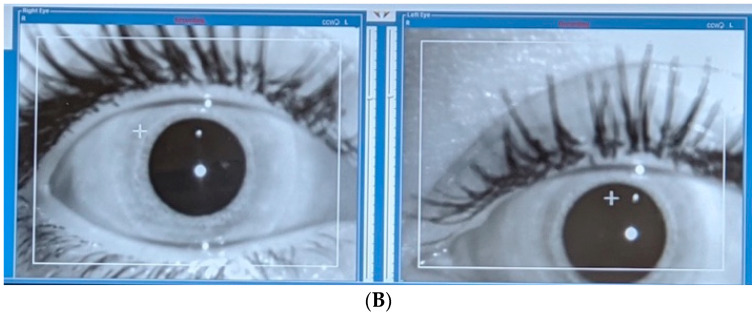
Pupillometry of both eyes. (**A**) Use of the pupillometry device. (**B**) Changes detected in the pupil, where the miosis and mydriasis described above are observed.

The pupil diameter can serve as a direct measure of the ANS and can be measured by automated pupillometry in real-time [17,19,21,40,41,42]. In this study, the pupil diameter was measured with the fully automated Vorteq^®^ system (Micromedical Technologies, Inc., Chatham, IL, USA). To control light affecting the pupil measures, the subjects wore goggles that covered both eyes to create a completely dark environment. When the eyes are in a dark environment, the parasympathetic activity is greatly reduced. If an increase in pupil diameter occurs (mydriasis), this would indicate an unopposed increase of the sympathetic nervous system [5,42,43]. The Vorteq^®^ system includes two infrared cameras that are built into the goggles. The system will directly and simultaneously measure the pupil diameter of both eyes (Figure 2).

Fully automated pupillometry devices to investigate responses of the autonomic nervous system have been used and validated previously in several studies [16,19,22,24,44,45,46,47,48,49]. The measurement error of automated pupillometry is minimal, and changes in pupil diameter less than 0.2 mm can be detected [47,49,50,51]. Both the pupillometry’s sensitivity and reliability to evaluate the autonomic nervous system have previously been shown [16,52,53,54]. Selva et al. [33] demonstrated previously that magnetic particles had a systemic influence on the ANS, making pupillometry an ideal tool to investigate subtle changes in the ANS.

### 2.4. Study Protocol

**Figure 3:**
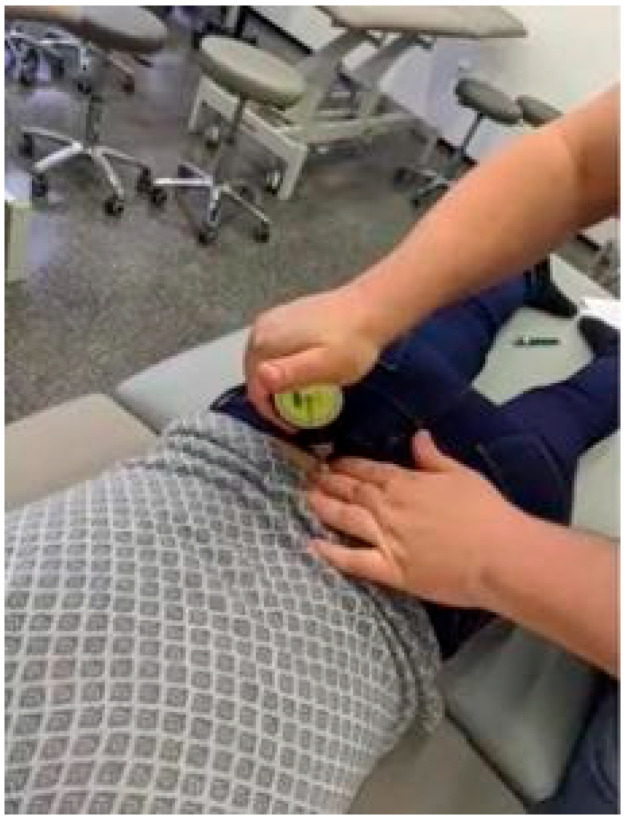
PA pressure on spine with algometer.

This study consisted of two measuring days in which each participant either received a placebo Kinesio tape or the TCMP (Magnetic Tape^®^, S.L., Valencia, Spain) intervention (both looked identical and could not be identified by the subject or researcher—physical therapist). Assignment of the intervention sequence was randomized, and the researcher applying the tape was not aware of which tape sample was the magnetic tape (the study tape was identified as tape 1 and tape 2). After providing consent to participate, the subject was positioned on a treatment table in the prone position. In this position, therapist 1 provided a 2 kg manual directed force posterior to anterior (PA) pressure on each spinous process through a pressure pain threshold (PPT) using a Wagner Force Dial FDK 20 algometer (Wagner Instruments, Greenwich, CT, USA) with a 1 cm^2^ and documented if this pressure would elicit any pain (Figure 3) (dichotomous variable).

**Figure 4:**
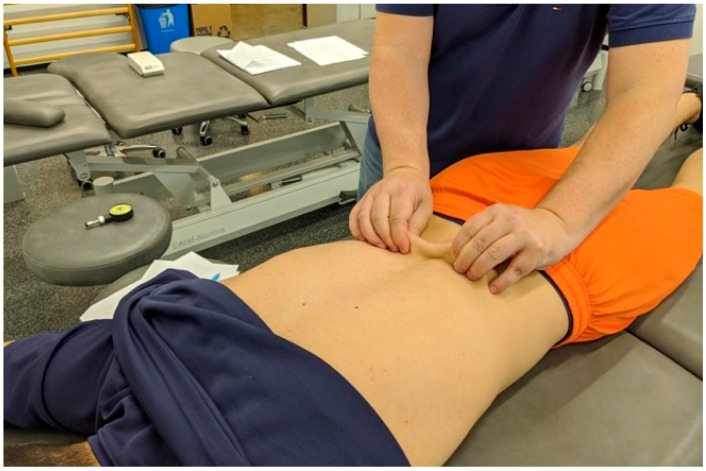
Segmental Skin Pinch Test.

The PA pressure was performed from S3 to C2 (Figure 3). After the PA assessment of the spine, therapist 1 performed the skin pinch test as previously described by Giamberardino et al. [55,56]. During this test, the skin of the back was pinched between the thumb and index finger at each spinal segment and distracted (S3 to C2) to see if this provoked pain (Figure 4).

Next, the subject moved to a second treatment table for the pupillometry/intervention part of this study. Another researcher (therapist 2) carried out this part of the study.

**Figure 5:**
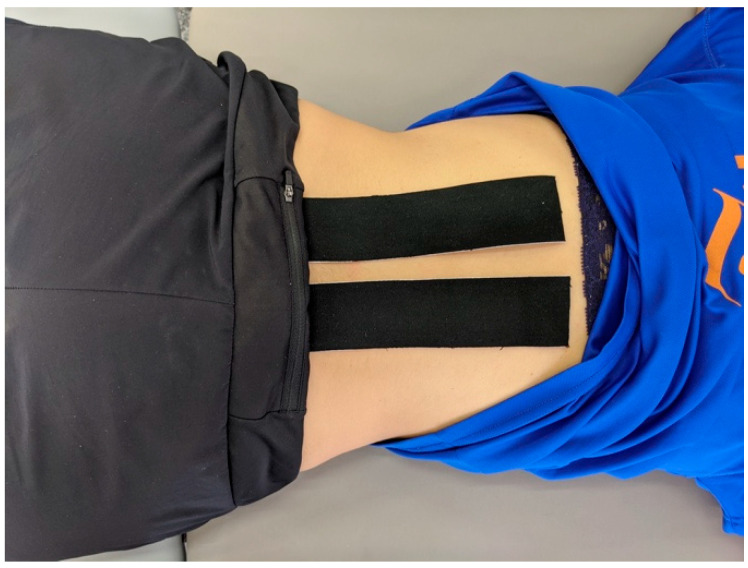
Lumbar Tape application.

Therapist 2 was blinded to the results of the PA pressure assessment. The TCMP was applied bilateral paravertebrally from the mid sacral to the T10 region (Figure 5).

Following the pupillometry protocol, the tape was removed, and the subject returned to therapist 1 for a post-intervention PA pain and skin pinch assessment. After this, the measurement phase of this part of the study was completed. On day 2 of the study, the subject underwent the same measurement protocol but received the control tape application (tape without magnetic particles).

### 2.5. Pupillometry Measurement Protocol

After accommodation to the darkness for two minutes, the pupil diameter was recorded continuously for a 60-s duration. Following the baseline measurement, the subject received the tape (experimental or control) application (Figure 5).

Directly following the tape placement, 60-s continuous pupil measurements of both eyes were recorded. After a 3-min period, the third and final 60-s pupil measurement was recorded. This same pupillometry measurement protocol was previously reported by Sillevis et al. [22]. The testing environment was temperature-controlled and remained the same for all subjects during this study.

### 2.6. Sample Size

The sample size was determined with the first 10 subjects recruited in the study, using a t-test for paired data on the immediate post-treatment pupillometry values on the first and second day between both types of tape. Accepting a risk α of 0.05, a power of 80% and losses of 20%, a sample of 25 was estimated.

### 2.7. Statistical Analysis

For the statistical analysis, the R Ver. 5.3.1 program was used (R Foundation for Statistical Computing, Institute for Statistics and Mathematics, Welthandelsplatz 1, 1020 Vienna, Austria). The level of significance was established at *p* < 0.05. The qualitative variables were described in absolute values and frequencies and the quantitative variables with mean and standard deviation. In the case of the outcome variables, the estimated marginal means adjusted by the baseline values with their standard errors (SE) as well as the average with 95% CI are shown. An analysis of covariance (ANCOVA) was used to determine the differences between both types of tape throughout the two treatment sessions with random subject effects, adjusting for baseline values at the start of each treatment session, according to the analysis of crossover studies proposed by Lawson [57]. Post hoc tests were performed between treatments with Bonferroni correction. In each model, the percentage of variance explained was evaluated with the adjusted R_a_^2^.

## 3. Results

The sample consisted of 22 patients, 14 women and 8 men, with an index of body mass of 25.42 ± 4.41 and an age of 44.59 ± 8.17 (Table 1).

Data expressed with mean ± standard deviation or with absolute and relative values (%).

### 3.1. Pupillometry Outcomes

The presence of significant differences between both tapes is checked for both the right eye (F(1) = 50.078, *p* ≤ 0.001) as in the left (F(1) = 26.371, *p* ≤ 0.001). In both cases the explained variance is moderate to high (R_a_^2^ = 0.818 and R_a_^2^ = 0.659, respectively) (Table 2).

Post hoc tests show that pupillometry values are lower with the TCMP compared to the control tape (86.328 (SE = 1.134) vs. 98.231 (SE = 1.134) in the right eye and 88.772 (SE = 2.069) vs. 104.698 (SE = 2.069) in left eye) and these differences are significant in both, the right (t(64) = 7.077, *p* ≤ 0.001) and the left eye (t(64) = 5.135, *p* ≤ 0.001) (Table 3).

**Figure 6:**
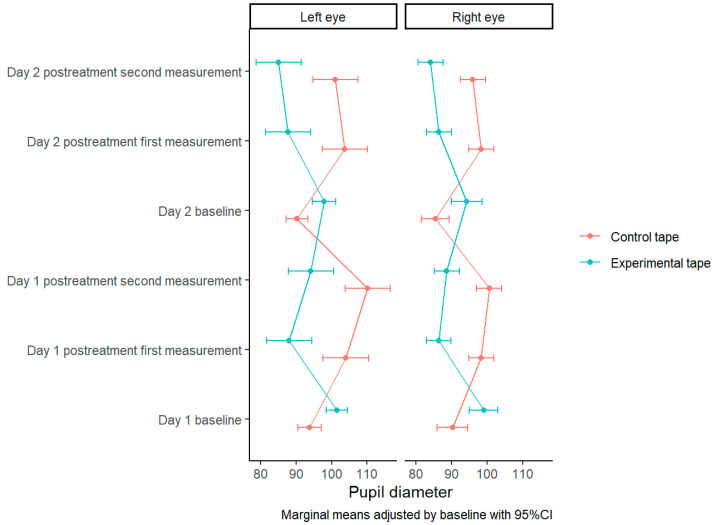
Pupillometry measurements.

It is verified how the pupillometry values decrease progressively with the experimental tape in both the right and left eyes while with the control tape, pupillometry increases through measurement time (Figure 6 and Supplementary Materials Table S1).

### 3.2. Pain with Posterior to Anterior Pressure on the Spine

There are no significant differences between both tapes at the cervical level (F(1) = 2.229, *p* = 0.137). The explained variance is moderate (R_a_^2^ = 0.445) (Table 4).

The post hoc test shows no significant differences between both tapes (t(250) = 1.493, *p* = 0.137) (Table 3). Pairwise comparisons show that the perceived pain hardly changed throughout the treatment sessions in both tapes (Supplementary Materials Table S2).

### 3.3. Pain with Paravertebral Skin Pinch Test on the Spine

The presence of significant differences between both tapes is checked in the right side at the thoracic level (F(1) = 7.933, *p* = 0.005), lumbar level (F(1) = 4.78, *p* = 0.03) and sacral level (F(1) = 5.576, *p* = 0.019). In all cases the explained variance is low (R_a_^2^ = 0.139, R_a_^2^ = 0.171 and R_a_^2^ = 0.23, respectively) (Table 4).

Post hoc tests show significant differences at overall pain values in thoracic (t(526) = 0.293, *p* = 0.005), lumbar (t(204) = 0.482, *p* = 0.03) and sacral (t(158) = 0.31, *p* = 0.019) right levels. These values are higher with the experimental tape compared to the control tape in thoracic [0.433 (SE = 0.073) vs. 0.14 (SE = 0.073)], lumbar [0.88 (SE = 0.155) vs. 0.398 (SE = 0.155)] and sacral [0.302 (SE = 0.092) vs. −0.008 (SE = 0.092)] right levels (Table 3).

**Figure 7:**
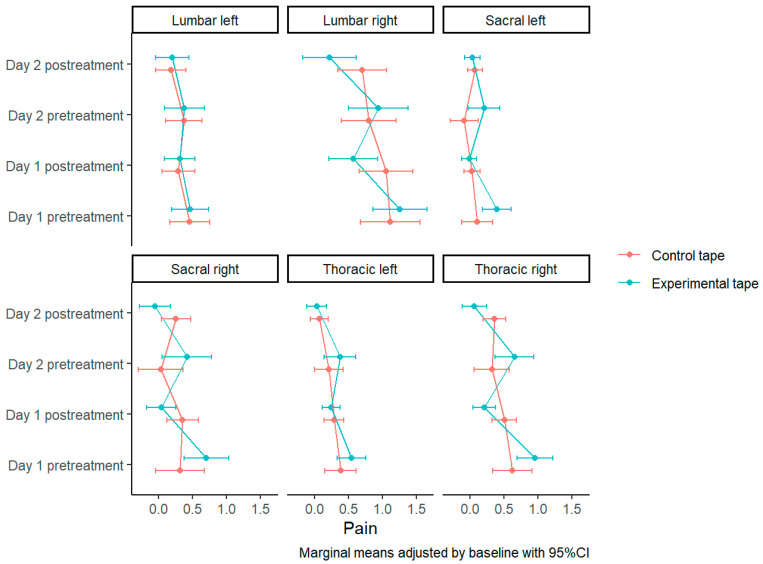
Skin Pinch Test Pain Measurements.

In the pairwise comparisons, it is evident how there is a decrease in perceived pain after the placement of both tapes, more accentuated in the experimental tape group, both at the right thoracic level (0.957 (SE = 0.133) to 0.214 (SE = 0.086) experimental vs. 0.623 (SE = 0.146) to 0.506 (SE = 0.093) control on the first day and 0.656 (SE = 0.146) to 0.066 (SE = 0.093) experimental vs. 0.322 (SE = 0.133) to 0.359 (SE = 0.086) control on the second day) and at the right lumbar level (1.261 (SE = 0.204) to 0.574 (SE = 0.182) experimental vs. 1.12 (SE = 0.222) to 1.056 (SE = 0.198) control on the first day and 0.94 (SE = 0.222) to 0.223 (SE = 0.198) experimental vs. 0.8 (SE = 0.204) to 0.704 (SE = 0.182) control the second day) and also at the right sacral level (0.701 (SE = 0.168) to 0.04 (SE = 0.108) experimental vs. 0.314 (SE = 0.183) to 0.35 (SE = 0.117) control on the first day and 0.414 (SE = 0.183) to -0.057 (SE = 0.117) experimental vs. 0.028 (SE = 0.168) to 0.253 (SE = 0.108) control the second day) (Figure 7 and Supplementary Materials Table S3).

## 4. Discussion

To evaluate the short-term effect of TCMP in subjects with lower back pain, the aim of this study was twofold. The primary aim was to investigate if paravertebral applied with magnetic particles containing tape in the low back region had a direct effect on the functioning of the ANS. The secondary aim was to investigate if the magnetic tape resulted in an immediate change in pain when posterior to anterior pressure was applied to the spinous process and during the segmental skin pinch test within this subject sample.

It has been demonstrated that changes in autonomic activity were significantly correlated with changes in subjective pain and prefrontal hemodynamic activity [23]. The ANS also has its action in the mediation of inflammatory pain [24].

It has been demonstrated that the pupil diameter can be used as a direct measure of the ANS function [17,19,21,40,41,42]. The pupil diameter is not static; it reflects the direct “live” balance between the two components of the ANS [5,43]. Consequently, it is necessary to capture the pupil for a more extended period. In this study, the pupil measurement duration was 60 s. This methodology should have minimized the direct effect of pupillary fluctuation and minimized the threat to the internal validity of this study. Pupillometry has been previously demonstrated to be a valid and reliable method of assessing the nervous system without much examiner bias [16,17,42,44,45,47,49,51,57,58]. When there is a noxious stimulus, the sympathetic systems create the dilation of the pupil through pathways that pass through the midbrain and the hypothalamus. This indicates a central supraspinal mechanism affecting the pupil diameter [59]. In complete darkness the parasympathetic nervous system’s activity is greatly reduced; therefore, the pupil diameter is a reflection of the relatively unopposed activity of the SNS [5,42,43].

The results of this study investigating the pupil response after the application of a TCMP targeting the paravertebral region of the sacral, lumbar and lower thoracic spine demonstrates that the mean pupil diameter in the placebo tape group significantly increased in diameter for both eyes. This would indicate a decrease in parasympathetic activity or an increase in activity of the sympathetic system. Such a difference seems to occur primarily between baseline and immediate post intervention and 3 min post-intervention measures. However, one must consider that the PA assessment during the pretest could have been provocative. The findings correlate with the fact that in the control group, more subjects reported an increase in painful segments with PA during post intervention testing.

In the lumbar spine, the number of segments increased from 19 painful segments to 30 painful segments. In the thoracic spine test, there was an increase from 17 to 25 painful segments. The application of the TCMP significantly decreased the pupil diameter in both eyes. This would indicate an increase in parasympathetic activity or a concurrent decrease in activity of the sympathetic system. Such a difference seems to occur primarily between baseline and immediately following the intervention and 3 min post-intervention measures. This seems directly related to the effect that the TCMP had on the ANS.

Persistent neuro-epidermal communication further appears to be coordinated by the sympathetic nervous system (SNS), whose sensory neuron cell bodies occupy dorsal root ganglia, innervating both the skin and CNS and the hypothalamic-pituitary-adrenal (HPA)-axis [18,50]. Interestingly, mammalian epidermal keratinocytes express all HPA-axis components, which function to regulate cutaneous anti-microbial defense [58].

The principal link between epidermal and neurodevelopment has further been observed to persist postnatally, with the notion of a skin-brain or brain-skin axis growing in popularity [58]. Epidermal keratinocytes appear to be central to this association, with these ‘information and sensory processing cells’ expressing numerous receptors found within the central nervous system (CNS) [58].

It is observed that the manipulation of the somatic elements of the thoracic spine did not produce changes in the diameter of the pupil [23], on the other hand, the epidermal action of TCMP did achieve it by regulating the ANS, therefore influencing the perceived pain. The importance and direct relationship of epidermal cells with pain has already been described [31]. The direct relationship of the epidermis with the thalamus has also been demonstrated, in addition to the influence of magnetic fields on keratinocytes [27,31]. Our findings appear to provide support for the theory that keratinocytes affect the free nerve endings in the epidermis, and that the firing of these nerves can result in a decreased activity at the spinal cord. Therefore, decreasing a state of sensitization.

In the study by Selva-Sarzo et al. [33], they also obtained a decrease in perceived pain and modulation of the vascularization of the lower limbs when performing superficial neuromodulation. Importantly, the brain, epidermis and skin appendages develop in synchronization, all originating from the embryonic ectoderm [58]. This embryological hierarchy is explained because the epidermis comes from the ectoderm and the somatic system from the mesoderm that was formed with cells from the ectoderm and endoderm. If we assume that what is created first is vital for life, the epidermis was generated from an earlier embryological layer than the somatic system. Our finding may indicate that the modulation of the epidermis is a priority for the ANS, and therefore for the CNS, rather than modulation of the somatic system to influence the organism systemically.

Epidermal modulation concurs with the findings of Hinman et al. [60], who reported a decrease in pain and improved range of motion in the knee following magnet application. The results also correlate with the findings of Alfano et al. [37], who demonstrated that there was a short-term benefit of magnets on pain reported by a group of fibromyalgia patients.

Pain is the result of spinal cord inter-neurons activity [4,9,59,60], which will determine the accumulative effect of the efferent input. This inter-neuron activity is not yet fully understood [61]. The results of this study would support that the TCMP directly affects inter-neural activity at the spinal segments influencing the ascending central and cortical pathways, (HPA)-axis influencing and in the thalamus.

It also appeared that the TCMP had an instant effect on the Skin Pinch Test with less painful segments reported. The fact that the tape was removed before therapist 1 re-examined the subject supports the thought that this change in activity by the keratinocytes is lasting longer than the time the tape is applied. Follow up studies should consider measuring the effect of nerve function and the long-term effect of the TCMP on the pain perception in subjects with pain.

Our results demonstrate physiological changes in the baseline state of the autonomic nervous system, implying a systemic response. This finding concurs with the finding of Selva-Sarzo et al. [33], who demonstrated that TCMP caused immediate changes in blood flow. This reinforced the hypothesis that a rapid systemic change can be created through epidermal stimulation. Therefore, it supports the hypothesis that magnetic therapy might provide a non-invasive, safe, and easy method to treat the area of pain directly [37]. Likewise, this therapy offers the potential to reduce the health care expenditures for managing chronic musculoskeletal disorders [38].

### Limitations and Future Directions

There were a few limitations to this study. First, the pupil diameter itself is by no means only pain specific [17,19]. It does appear as though the pupil diameter is an indication for general arousal, stress, anxiety and noxious stimulation [62,63]. We cannot identify if any of these factors affected the outcomes of this study. However, since the pupil measures were taken immediately following the application of the tape, the pupillometry findings should be a direct reflection of the change in autonomic functioning. Secondly, the subjects were randomly assigned to receive either the control or the TCMP first. The results of this study support the premise that the tape directly affects the functioning of the keratinocytes. What cannot be determined is if the subjects that received the TCMP first had a carry-over effect into the second day of testing when the placebo tape was used. Furthermore, a final limitation is that this was a study with only 22 subjects. Therefore, the need for similar studies in a larger group of patients is suggested. An unequal number of male and female subjects with an age range between 26 and 58 could have negatively affected the pupil response. Based on these limitations, the generalizability of the results is limited. We postulate that this modulation by TCMP has an immediate effect on the autonomic system and reducing perceived pain, opening a large field of future research.

## 5. Conclusions

This study demonstrates that TCMP has an immediate effect on the functioning of the ANS, resulting in a decrease in overall pain in subjects with lower back pain. It was demonstrated that TCMP applied to the lower back results in an immediate short-term reduction of pain with spinal posterior-anterior applied force and the paravertebral skin pinch test. Future research is necessary to evaluate the long-term effects of TCMP on pain and range of motion. Additionally, the new paradigm offered here is expected to become a source of new questions regarding the benefit of TCMP applied on the epidermis, leading to future research. Due to the limited sample size of the present study, the conclusions presented are limited.

## Figures and Tables

**Table 1:** Baseline characteristics of the participants.

*n*		22
Gender, *n* (%)	Female	14 (63.6)
	Male	8 (36.4)
Age		44.59 ± 8.17
Weight (kg)		72.41 ± 12.67
Height (cm)		168.91 ± 8.90
Body mass index		25.42 ± 4.41

**Table 2:** Pupillometry analysis model.

		Coefficient (SE)	95% CI	t Value	^a^ *p* Value	F(df)	^a^ *p* Value	Overall Model
Right eye	(Intercept)	29.694 (SE = 7.26)	15.19, 44.198	4.090	<0.001	16.727(1)	<0.001	R_a_^2^ = 0.818
	Day 1 postreatment first measurement	−0.014 (SE = 1.336)	−2.682, 2.655	−0.010	0.992	1.442(3)	0.239	F = 16.113, *p* < 0.001
	Day 1 postreatment second measurement	2.27 (SE = 1.336)	−0.399, 4.938	1.699	0.094		NA	
	Day 2 postreatment first measurement	0.034 (SE = 1.336)	−2.634, 2.702	0.025	0.98		NA	
	Treatment	5.952 (SE = 0.841)	4.271, 7.632	7.077	<0.001	50.078(1)	<0.001	
Left eye	(Intercept)	30.239 (SE = 17.718)	−5.157, 65.635	1.707	0.093	2.913(1)	0.093	R_a_^2^ = 0.659
	Day 1 postreatment first measurement	−0.717 (SE = 2.403)	−5.516, 4.083	−0.298	0.766	1.9(3)	0.138	F = 7.527, *p* < 0.001
	Day 1 postreatment second measurement	5.411 (SE = 2.403)	0.612, 10.211	2.252	0.028		NA	
	Day 2 postreatment first measurement	−1.021 (SE = 2.403)	−5.82, 3.779	−0.425	0.672		NA	
	Treatment	7.963 (SE = 1.551)	4.865, 11.061	5.135	<0.001	26.371(1)	<0.001	

95% CI: 95% confidence interval; SE: standard error; F(df): F statistic (degrees of freedom). Contrast day measurement against Day 2 posttreatment second measurement. ^a^ significant if *p* < 0.05 (shown in red).

**Table 3:** Experimental and control tape adjusted marginal means by baseline and pairwise comparison.

		Marginal Means Adjusted by Baseline (SE)	95% CI		Marginal Means Difference and *p* Value ^a^
Pupillometry					
Right eye	Control tape	98.231 (SE = 1.134)	95.965, 100.498	Control tape—Experimental tape	11.903 (SE = 1.682)
	Experimental tape	86.328 (SE = 1.134)	84.062, 88.595		t(64) = 7.077, *p* ≤ 0.001
Left eye	Control tape	104.698 (SE = 2.069)	100.565, 108.832	Control tape—Experimental tape	15.926 (SE = 3.101)
	Experimental tape	88.772 (SE = 2.069)	84.638, 92.906		t(64) = 5.135, *p* ≤ 0.001
Posterior-anterior directed pressure					
	Control tape	0.163 (SE = 0.051)	0.062, 0.264	Control tape—Experimental tape	0.109 (SE = 0.073)
	Experimental tape	0.054 (SE = 0.051)	−0.046, 0.155	Control tape—Experimental tape	t(250) = 1.493, *p* = 0.137
Paravertebral skin pinch test					
Thoracic right	Control tape	0.433 (SE = 0.073)	0.289, 0.576	Control tape—Experimental tape	0.293 (SE = 0.104)
	Experimental tape	0.14 (SE = 0.073)	−0.003, 0.283		t(526) = 0.293, *p* = 0.005
Lumbar right	Control tape	0.88 (SE = 0.155)	0.574, 1.186	Control tape—Experimental tape	0.482 (SE = 0.22)
	Experimental tape	0.398 (SE = 0.155)	0.092, 0.704		t(204) = 0.482, *p* = 0.03
Sacral right	Control tape	0.302 (SE = 0.092)	0.12, 0.483	Control tape—Experimental tape	0.31 (SE = 0.131)
	Experimental tape	−0.008 (SE = 0.092)	−0.19, 0.173		t(158) = 0.31, *p* = 0.019
Thoracic left	Control tape	0.183 (SE = 0.057)	0.072, 0.295	Control tape—Experimental tape	0.041 (SE = 0.081)
	Experimental tape	0.143 (SE = 0.057)	0.031, 0.254		t(526) = 0.041, *p* = 0.614
Lumbar left	Control tape	0.234 (SE = 0.096)	0.044, 0.424	Control tape—Experimental tape	−0.019 (SE = 0.137)
	Experimental tape	0.253 (SE = 0.096)	0.063, 0.443		t(204) = −0.019, *p* = 0.891
Sacral left	Control tape	0.052 (SE = 0.047)	−0.04, 0.144	Control tape—Experimental tape	0.039 (SE = 0.067)
	Experimental tape	0.013 (SE = 0.047)	−0.079, 0.105		t(158) = 0.039, *p* = 0.557

95% CI: 95% confidence interval; SE: standard error; t(df): t statistic (degrees of freedom). ^a^ significant if *p* < 0.05 (shown in red).

**Table 4:** Pain analysis models.

		Coefficient (SE)	95% CI	F(df)	^a^ *p* Value	Overall Model
Posterior-anterior directed pressure						
	(Intercept)	0.051 (SE = 0.036)	−0.021, 0.122	1.921(1)	0.167	R_a_^2^ = 0.445
	Period	0.107 (SE = 0.036)	0.036, 0.179	8.768(1)	0.003	F = 9.805, *p* ≤ 0.001
	Treatment	0.054 (SE = 0.036)	−0.017, 0.126	2.229(1)	0.137	
Paravertebral skin pinch test						
Thoracic right	(Intercept)	0.153 (SE = 0.054)	0.046, 0.26	7.956(1)	0.005	R_a_^2^ = 0.139
	Period	0.074 (SE = 0.052)	−0.028, 0.176	2.025(1)	0.155	F = 4.546, *p* ≤ 0.001
	Treatment	0.146 (SE = 0.052)	0.044, 0.248	7.933(1)	0.005	
Lumbar right	(Intercept)	0.486 (SE = 0.127)	0.236, 0.735	14.71(1)	<0.001	R_a_^2^ = 0.171
	Period	0.176 (SE = 0.111)	−0.042, 0.394	2.527(1)	0.113	F = 2.891, *p* ≤ 0.001
	Treatment	0.241 (SE = 0.11)	0.024, 0.458	4.78(1)	0.03	
Sacral right	(Intercept)	0.031 (SE = 0.067)	−0.101, 0.164	0.221(1)	0.639	R_a_^2^ = 0.23
	Period	0.048 (SE = 0.065)	−0.081, 0.177	0.549(1)	0.46	F = 3.191, *p* ≤ 0.001
	Treatment	0.155 (SE = 0.066)	0.025, 0.285	5.576(1)	0.019	
Thoracic left	(Intercept)	0.077 (SE = 0.041)	−0.004, 0.158	3.451(1)	0.064	R_a_^2^ = 0.153
	Period	0.108 (SE = 0.04)	0.028, 0.187	7.111(1)	0.008	F = 4.994, *p* ≤ 0.001
	Treatment	0.02 (SE = 0.04)	−0.059, 0.1	0.255(1)	0.614	
Lumbar left	(Intercept)	0.21 (SE = 0.072)	0.068, 0.352	8.516(1)	0.004	R_a_^2^ = 0.146
	Period	0.056 (SE = 0.069)	−0.079, 0.191	0.672(1)	0.413	F = 2.568, *p* ≤ 0.001
	Treatment	−0.009 (SE = 0.068)	−0.144, 0.126	0.019(1)	0.891	
Sacral left	(Intercept)	0.043 (SE = 0.033)	−0.023, 0.108	1.66(1)	0.2	R_a_^2^ = 0.008
	Period	−0.023 (SE = 0.033)	−0.088, 0.042	0.477(1)	0.491	F = 1.057, *p* = 0.398
	Treatment	0.02 (SE = 0.033)	−0.046, 0.085	0.347(1)	0.557	

95% CI: 95% confidence interval; SE: standard error; F(df): F statistic (degrees of freedom). ^a^ significant if *p* < 0.05 (shown in red).

## Data Availability

The data presented in this study are available on request from the corresponding authors. The data are not publicly available due to ethical restrictions.

## Supplementary Materials

The following supporting information can be downloaded at: https://www.mdpi.com/article/10.3390/biomedicines11061551/s1, Table S1: Marginal means adjusted by baseline pupillometry outcomes; Table S2: Marginal means adjusted by baseline posterior-anterior directed pressure test outcomes.; Table S3: Marginal means adjusted by baseline paravertebral skin pinch test outcomes; Table S4: Marginal means adjusted by baseline paravertebral skin pinch test outcomes.
