# Supplementary material for: Neuromodulation of the Autonomic Nervous System in Chronic Low Back Pain: A Randomized, Controlled, Crossover Clinical Trial
Source: Biomedicines. 2023 May 26;11(6):1551. doi: 10.3390/biomedicines11061551 (PMC10295027; doi:10.3390/biomedicines11061551)
Supplement: Supplementary file 1 [file biomedicines-11-01551-s001.zip › biomedicines-2407269-supplementary.pdf]

Supplementary Materials

Table S1. Marginal means adjusted by baseline pupillometry outcomes.

			Marginal means (SE)	95%CI
Right eye	Day 1 baseline	Control tape	90.166 (SE = 2.157)	85.86, 94.472
		Experimental tape	98.917 (SE = 1.976)	94.973, 102.862
Left eye		Control tape	93.827 (SE = 1.65)	90.533, 97.12
		Experimental tape	101.462 (SE = 1.512)	98.445, 104.479
Right eye	Day 1 postreatment first measurement	Control tape	98.218 (SE = 1.76)	94.702, 101.734
		Experimental tape	86.315 (SE = 1.745)	82.829, 89.8
Left eye		Control tape	103.982 (SE = 3.176)	97.636, 110.327
		Experimental tape	88.055 (SE = 3.165)	81.732, 94.378
Right eye	Day 1 postreatment second measurement	Control tape	100.501 (SE = 1.76)	96.985, 104.017
		Experimental tape	88.598 (SE = 1.745)	85.112, 92.083
Left eye		Control tape	110.11 (SE = 3.176)	103.764, 116.456
		Experimental tape	94.183 (SE = 3.165)	87.86, 100.507
Right eye	Day 2 baseline	Control tape	85.425 (SE = 1.976)	81.481, 89.37
		Experimental tape	94.177 (SE = 2.157)	89.871, 98.483
Left eye		Control tape	90.261 (SE = 1.512)	87.244, 93.278
		Experimental tape	97.897 (SE = 1.65)	94.603, 101.19
Right eye	Day 2 postreatment first measurement	Control tape	98.265 (SE = 1.745)	94.78, 101.751
		Experimental tape	86.362 (SE = 1.76)	82.846, 89.878
Left eye		Control tape	103.678 (SE = 3.165)	97.355, 110.001
		Experimental tape	87.751 (SE = 3.176)	81.406, 94.097
Right eye	Day 2 postreatment second measurement	Control tape	95.941 (SE = 1.745)	92.456, 99.427
		Experimental tape	84.038 (SE = 1.76)	80.522, 87.554
Left eye		Control tape	101.025 (SE = 3.165)	94.702, 107.348
		Experimental tape	85.098 (SE = 3.176)	78.753, 91.444

95%CI: 95% confidence interval; SE: Standard error.

Table S2. Marginal means adjusted by baseline posterior-anterior directed pressure test outcomes.

		Marginal means (SE)	95%CI
Day 1 pretreatment	Control tape	0.114 (SE = 0.172)	-0.243, 0.47
	Experimental tape	0.252 (SE = 0.187)	-0.137, 0.642
Day 1 postreatment	Control tape	0.174 (SE = 0.09)	-0.014, 0.362
	Experimental tape	0.239 (SE = 0.099)	0.033, 0.446
Day 2 pretreatment	Control tape	0.052 (SE = 0.187)	-0.337, 0.442
	Experimental tape	0.191 (SE = 0.172)	-0.166, 0.547
Day 2 postreatment	Control tape	-0.022 (SE = 0.099)	-0.228, 0.185
	Experimental tape	0.043 (SE = 0.09)	-0.145, 0.232

95%CI: 95% confidence interval; SE: Standard error.

Table S3. Marginal means adjusted by baseline paravertebral skin pinch test outcomes.

		Marginal means (SE)	95%CI
Day 1 pretreatment	Control tape	0.252 (SE = 0.106)	0.043, 0.462
	Experimental tape	0.114 (SE = 0.097)	-0.078, 0.306
Day 1 postreatment	Control tape	0.27 (SE = 0.065)	0.142, 0.399
	Experimental tape	0.162 (SE = 0.06)	0.044, 0.28
Day 2 pretreatment	Control tape	0.191 (SE = 0.097)	-0.001, 0.383
	Experimental tape	0.052 (SE = 0.106)	-0.157, 0.262
Day 2 postreatment	Control tape	0.056 (SE = 0.06)	-0.062, 0.173
	Experimental tape	-0.053 (SE = 0.065)	-0.182, 0.076

95%CI: 95% confidence interval; SE: Standard error.

Table S4. Marginal means adjusted by baseline paravertebral skin pinch test outcomes.

			Marginal means (SE)	95%CI
Thoracic right	Day 1 pretreatment	Control tape	0.623 (SE = 0.146)	0.337, 0.909
		Experimental tape	0.957 (SE = 0.133)	0.695, 1.219
Lumbar right		Control tape	1.12 (SE = 0.222)	0.682, 1.559
		Experimental tape	1.261 (SE = 0.204)	0.859, 1.663
Sacral right		Control tape	0.314 (SE = 0.183)	-0.047, 0.676
		Experimental tape	0.701 (SE = 0.168)	0.37, 1.032
Thoracic left	Day 1 pretreatment	Control tape	0.386 (SE = 0.117)	0.156, 0.617
		Experimental tape	0.549 (SE = 0.107)	0.338, 0.76
Lumbar left		Control tape	0.457 (SE = 0.15)	0.163, 0.752
		Experimental tape	0.464 (SE = 0.137)	0.193, 0.734
Sacral left		Control tape	0.108 (SE = 0.116)	-0.121, 0.336
		Experimental tape	0.398 (SE = 0.106)	0.189, 0.607
Thoracic right	Day 1 postreatment	Control tape	0.506 (SE = 0.093)	0.323, 0.689
		Experimental tape	0.214 (SE = 0.086)	0.045, 0.382
Lumbar right		Control tape	1.056 (SE = 0.198)	0.665, 1.447
		Experimental tape	0.574 (SE = 0.182)	0.215, 0.933
Sacral right		Control tape	0.35 (SE = 0.117)	0.119, 0.581
		Experimental tape	0.04 (SE = 0.108)	-0.174, 0.254
Thoracic left	Day 1 postreatment	Control tape	0.291 (SE = 0.073)	0.148, 0.434
		Experimental tape	0.25 (SE = 0.067)	0.119, 0.382
Lumbar left		Control tape	0.29 (SE = 0.123)	0.047, 0.534
		Experimental tape	0.309 (SE = 0.113)	0.086, 0.532
Sacral left		Control tape	0.029 (SE = 0.059)	-0.087, 0.146
		Experimental tape	-0.01 (SE = 0.055)	-0.118, 0.099
Thoracic right	Day 2 pretreatment	Control tape	0.322 (SE = 0.133)	0.06, 0.584
		Experimental tape	0.656 (SE = 0.146)	0.37, 0.942
Lumbar right		Control tape	0.8 (SE = 0.204)	0.398, 1.201
		Experimental tape	0.94 (SE = 0.222)	0.502, 1.379
Sacral right		Control tape	0.028 (SE = 0.168)	-0.303, 0.359
		Experimental tape	0.414 (SE = 0.183)	0.053, 0.776
Thoracic left	Day 2 pretreatment	Control tape	0.216 (SE = 0.107)	0.005, 0.426
		Experimental tape	0.378 (SE = 0.117)	0.148, 0.608
Lumbar left		Control tape	0.371 (SE = 0.137)	0.101, 0.641
		Experimental tape	0.377 (SE = 0.15)	0.083, 0.672
Sacral left		Control tape	-0.083 (SE = 0.106)	-0.292, 0.127
		Experimental tape	0.208 (SE = 0.116)	-0.021, 0.436
Thoracic right	Day 2 postreatment	Control tape	0.359 (SE = 0.086)	0.19, 0.527
		Experimental tape	0.066 (SE = 0.093)	-0.117, 0.249
Lumbar right		Control tape	0.704 (SE = 0.182)	0.345, 1.064
		Experimental tape	0.223 (SE = 0.198)	-0.169, 0.614
Sacral right		Control tape	0.253 (SE = 0.108)	0.039, 0.467
		Experimental tape	-0.057 (SE = 0.117)	-0.287, 0.174
Thoracic left	Day 2 postreatment	Control tape	0.076 (SE = 0.067)	-0.055, 0.207
		Experimental tape	0.035 (SE = 0.073)	-0.108, 0.178
Lumbar left		Control tape	0.178 (SE = 0.113)	-0.045, 0.401
		Experimental tape	0.197 (SE = 0.123)	-0.047, 0.44
Sacral left		Control tape	0.075 (SE = 0.055)	-0.033, 0.183
		Experimental tape	0.036 (SE = 0.059)	-0.081, 0.152

95%CI: 95% confidence interval; SE: Standard error.
